# Spermatic Colic

**Published:** 1874-12

**Authors:** 


ON SPERMATIC COLIC.—Reliquet insists much on these facts in making the diagnosis: 1. That the first pain is produced suddenly at the time of coitus. 2. That subsequently every erection, or even every desire for coitus, produces pain. They are, he says, the subjective symptoms. These, combined with the objective signs, especially with the uniform swelling of the seminal vesicle, should lead to a correct diagnosis. Reliquet relates, in the *Gazette des Hopitaux*, September 3, 1874, the case of a hairdresser, aged thirty-five, married, pale, thin, delicate, and exhausted-looking, who consulted the author on March 12, 1874. For several years he had often suffered, when at stool, from the emission of a small quantity of thick white liquid. For two months he had experienced pain, extending from the anus to the perineum, or emissions during coitus or when emptying the bowels. The erect posture and walking provoked frequent micturition. Attempts to retain his urine caused acute pain and cold sweats. The pain lasted four or five minutes after urination. He lost desire to sleep. Venereal desires and erection provoked pain. During one month and a half he had passed small quantities of blood with his urine.

On March 12 a gum elastic bougie (French, No. 20,) was passed easily into the bladder, but caused excessive pain in the deep part of the urethra. He was sounded for stone the next day, but no foreign body was discovered in the bladder. On examination from the rectum, the left vesicula seminalis was found swollen, hard and smooth. It was painful on pressure. After the sounding, an attempt was made to pass the bougie once more, but the instrument was arrested at the neck of the bulb, and then withdrawn. After two or three minutes of extremely sharp cutting pains in the penis and the anus, the patient stooped down, and, emptying the bladder, expelled as many as forty small opaque white bodies, about the size of a small pin's head or a lentil. They presented facettes and blunt angles, like prostatic calculi, but their consistence was soft, and they could be crushed between the fingers. To the naked eye they appeared to be formed of a white homogeneous material, devoid of any capsule.

After the passage of these bodies, the symptoms quickly subsided, and, on examining from the rectum on the third day, the left seminal vesicle was found of the same size as the right.

On March 27 the man had returned to his usual occupations, and only had to pass urine twice in the night.

Professor Robin, who examined the concretions, sent M. Reliquet the following report: The concretions, opaline, transparent, had a diameter of from one to two millimetres. Their substance, under the microscope, was marked by very fine and very short rectilinear striæ, parallel, closely approximated to each other. Acetic acid caused the striated condition to disappear, and rendered the substance quite translucent and homogeneous. At the same time it demonstrated numerous spermatozoids rolled together in the concretions formerly marked by the striæ. Where the striation was absent or scarcely marked on the concretions, which were of various forms, such as M. Robin has shown in the vesiculæ seminales of many healthy individuals (Charles Robin, *Lecons sur les Humeurs*, Paris, 1874, 2d edition, p. 443), these bodies had the aspect of normal ones. They had similar reactions to acetic acid, reactions which were not only those of mucus. Their volume was enormous, compared with that of the concretions in normal vesiculæ, and their consistence a little greater. But they only represented normal corpuscles, which had accidentally attained gigantic proportions, and englobed the spermatozoids in the usual way.

